# Signaling Pathways of the Acquired Immune System and Myocardial Dysfunction in Chronic Kidney Disease—What Do We Know So Far?

**DOI:** 10.3390/biom16010049

**Published:** 2025-12-29

**Authors:** Anila Duni, Christos Georgopoulos, Athanasios Kitsos, Georgios Markopoulos, Lefkothea Dova, Georgios Vartholomatos, Evangelia Dounousi

**Affiliations:** 1 Department of Nephrology, Faculty of Medicine, School of Health Sciences, University Hospital of Ioannina, University of Ioannina, 45500 Ioannina, Greece; georgopoulosch@gmail.com (C.G.); thkitsos@hotmail.com (A.K.); evangeldou@gmail.com (E.D.); 2 Laboratory of Hematology-Unit of Molecular Biology and Translational Flow Cytometry, University Hospital of Ioannina, 45500 Ioannina, Greece; geomarkop@gmail.com (G.M.); thea_dova@yahoo.gr (L.D.); gvarthol@gmail.com (G.V.)

**Keywords:** myocardial remodeling, myocardial fibrosis, myocardial strain, chronic kidney disease, CD4^+^ T cells, CD8^+^ T cells, regulatory T cells, B lymphocytes

## Abstract

Aberrant signaling pathways of the acquired immune system are implicated in the development of cardiovascular disease (CVD) and chronic kidney disease (CKD) phenotypes. Understanding the complex abnormalities of lymphocyte subpopulations in CKD is a prerequisite for elucidating their implication in uremic cardiomyopathy. T cell subsets display various patterns of association with indices of myocardial function in both experimental and clinical CKD models. The role of Tregs in CVD and CKD has attracted significant research interest. Although experimental data suggest a protective role of Tregs from the development of arterial hypertension- and pressure overload-induced myocardial hypertrophy, there might be a change in the regulatory T cell (Treg) phenotype towards a profibrotic one in the settings of CKD and heart failure. Depletion of B lymphocytes is a hallmark of CKD and heart failure, bearing adverse prognostic significance, yet evidence of B lymphocytes’ involvement in the pathogenesis of myocardial damage is currently lacking. Considering that myocardial remodeling is the final outcome of diverse pathogenic processes targeting the heart, the aim of this review is to present the evidence available up to now regarding the role of acquired immune cells in the pathogenesis of the structural and functional alterations of the myocardium in CKD.

## 1. Introduction

The chronic inflammatory state, as a chronic kidney disease (CKD) hallmark, is mediated by a complex interplay of cellular components of the innate and adaptive immune systems, related immune mediators and intertwining molecular signaling pathways [1,2]. On the other hand, a significant body of experimental and human research data indicate that immune mechanisms are implicated in all aspects of cardiovascular disease (CVD) phenotypes, including atherosclerosis and heart failure [3]. Cardiovascular disease and CKD are tightly interrelated with CKD patients, displaying a significant burden of cardiovascular morbidity and mortality [4]. Notably, myocardial remodeling, manifesting as fibrosis, left ventricular hypertrophy (LVH) and disordered myocardial strain, has already become evident during the early stages of CKD [5]. During the last two decades, the term cardiorenal syndrome (CRS) has been introduced and established to describe the cardiorenal interaction, when acute or chronic dysfunction of the heart or kidneys might lead to acute or chronic impairment in the function of the other organ [6]. Despite the reverse remodeling process developing following kidney transplantation, subclinical abnormalities in the biventricular strain may be observed in kidney transplant recipients (KTRs) as well [7].

Abundant accumulating evidence has unraveled the adverse and decisive role of the innate immune responses in the pathogenesis of CVD in patients with CKD [8]. However, there are scarce data regarding the involvement of the cellular components and related signaling pathways of the acquired immune system in the development of uremic cardiomyopathy. In the light of promising evidence from clinical trials targeting the adaptive immune cells in patients with atherosclerosis and trials of interleukin-1 (Il-1) and interleukin-6 (Il-6) signaling inhibition in patients with atherosclerosis and CKD, ongoing research shall pave the way for the development of therapeutic strategies that address the immune components in uremic cardiomyopathy [9,10,11].

Considering that the process of myocardial remodeling is the final shared outcome of diverse pathogenic processes targeting the heart, we will subsequently present the evidence available up to now regarding the role of acquired immune cells in the pathogenesis of the structural and functional alterations of the myocardium in CKD. Furthermore, we will attempt to identify similarities between uremic cardiomyopathy and other cardiomyopathy models with regard to the patterns of involvement of the acquired immune system cellular components, as a means to draw conclusions and create a foundation for future research. Accordingly, CKD and the related uremic environment might also well represent a specific injurious process to the myocardium, which in this setting undergoes a spectrum of changes from adaptive to maladaptive hypertrophy. Over and above that, the identification of specific and unique immunological traits and clinical parameters that provide the necessary background for the development of uremic cardiomyopathy in CKD patients and KTRs would be of paramount importance.

## 2. Alterations in the Cellular Components of the Acquired Immune System in CKD

### 2.1. T Lymphocytes

A detailed description of the alterations in the components of the acquired immune system in CKD is beyond the scope of this review article and has been extensively addressed by previous excellent reviews [12,13,14,15,16]. Nevertheless, acknowledging and understanding the abnormalities that characterize the acquired immune system status in these patients is a prerequisite to further clarify the implication of immune mechanisms in the development of uremic cardiomyopathy. Patients with advanced CKD and kidney failure display a progressive decline in circulating T cell count together with significant alterations in T cell subset constitution and activation status, including reduced CD4-to-CD8 ratio, increased T helper 1 (Th1)-to-T helper 2 (Th2) ratio and exhaustion of naïve and central memory CD4^+^ and CD8^+^ T cells [12,13,14,15]. Available evidence supports the interplay of a constellation of factors underlying the aberrant expression of naïve and memory T cells in these patients, such as the uremic milieu, the amplification of oxidative stress and the persistent microinflammatory state, as well as dialysis related factors, among others [12,13,14,15]. Various mechanisms appear to be involved in the pathogenesis of the phenotypic and functional alterations of T cell subsets in CKD patients. Accordingly, compared with healthy individuals, the thymic output of naïve T cells, as indicated by the amount of the genomic DNA remnants produced during T cell receptor (TCR) rearrangements or otherwise TCR excision circles, appears to be substantially decreased in kidney failure patients regardless of age [17]. Data from flow cytometry analyses have revealed that there is a high percentage of T lymphocytes expressing activation and apoptosis markers simultaneously in CKD patients and to a greater extent in those undergoing renal replacement therapy (RRT). Accordingly, naïve T cells have been found to display enhanced expression of CD69; CD25, which is the IL-2 receptor; the inflammatory chemokine receptors CXCR3 and CCR5; as well as annexin and Fas (CD95) [14,18,19]. These T lymphocytes are susceptible to activation-induced apoptosis after stimulation with phytohemagglutinin or anti-CD3 [18]. Of note, enhanced apoptosis mainly affects the naïve and the central memory T lymphocytes, but not the effector memory T lymphocytes [18]. Furthermore, according to early studies, antigen recognition by T cells appears to be affected in CKD patients both due to decreased TCR expression as well as defective expression and binding of costimulatory ligands such as CD86 with CD28 on T lymphocytes [20,21]. Thus, in vitro data have shown that incubation of CD4^+^ T lymphocyte cultures from healthy individuals and from hemodialysis patients is associated with markedly decreased TCR density on the CD4^+^ T lymphocytes [20,21]. Immunophenotyping of T cells from patients with non-end-stage CKD and kidney failure patients has shown increased frequency of both exhausted and anergic CD4^+^ T cells and CD8^+^ T cells, a state which has been ascribed to chronic stimulation in the setting of persistent inflammation [22]. Notably, patients with kidney failure display increased counts of terminally differentiated CD4^+^ and CD8^+^ T cells which do not express the costimulatory molecule CD28 and which possess pronounced proinflammatory and cytotoxic features [15,23].

T cell abnormalities persist following kidney transplantation and apart from the immunosuppressive regimen, several other potential culprit mechanisms have been suggested, including uremia-induced irreversible T cell phenotype defects in the setting of a long dialysis vintage as well as reduced thymic emigration [14,24,25,26,27]. Accumulating evidence indicates that similar to patients with kidney failure, increased levels of CD28null CD4^+^ and CD8^+^ T cells together with lower effector memory T cells have been observed in kidney transplant recipients and have been associated with adverse graft outcomes [28,29,30].

### 2.2. T Regulatory Cells

Regulatory T cells (Tregs) include naturally derived Tregs, also known as CD4^+^CD25^+^ forkhead/winged helix transcription factor (Foxp3) (+); Treg cells which maturate in the thymus; and adapted Tregs which derive from the differentiation of naïve CD4^+^ T cells induced by signaling pathways involving cytokines or antigen stimulation [31]. The essential role of Tregs in the regulation of immune homeostasis and in the suppression of autoimmune inflammatory responses has been well established. Therefore, the protective effects of Tregs via TGF-β- and IL-10-mediated pathways have come to the spotlight as potential therapeutic targets for several pathogenic processes, including models of CKD and kidney transplantation [31,32,33]. CKD is associated with alterations in Treg populations; however, additional research is needed to further clarify potential ambiguous points in the currently available clinical studies, including heterogeneities in patient characteristics and protocol design. Notably, incubation of isolated Tregs from healthy individuals with uremic serum from hemodialysis patients led to a decline in the Treg number together with the downregulation of FOXP3 expression and an impaired suppressive capacity of these cells, thus highlighting the pathogenic implication of the uremic milieu [31,32,33]. Augmented Treg apoptosis rates and diminished inhibitory capacities for phytohemagglutinin-induced proliferation of CD4^+^ T cells have been demonstrated in patients with advanced CKD as well as in patients undergoing peritoneal dialysis (PD) and hemodialysis [34]. CD4^+^ T cells isolated from the whole blood of hemodialysis patients compared to those isolated from healthy controls display significantly lower proportions of CD4^+^CD25^+^FOXP3^+^ Tregs and CD4^+^GATA3^+^ Th2 cells as well as lower Il-4 levels when submitted to antigen-independent T cell mitogen stimulation in vitro [35]. These data further reinforce the available evidence regarding the suppressed anti-inflammatory status of Tregs and Th2 cells in advanced CKD. Simultaneously, hemodialysis patients exhibit an imbalance between the Treg number and function and the proinflammatory Th17 CD4^+^ T cells, which manifests as increased peripheral Th17 cell counts together with accentuated expression of IL-17, IL-6 and IL-23 as well as of the DNA binding transcription factor RAR-related orphan receptor gamma (RORγ) RNA [36]. Lower inducibility of Il-2 in kidney failure as well as upregulation of both IL-6 and TGF-β, which further induce effector Th17 cells, lead to the inhibition of the CD4^+^CD25^+^FoxP3^+^ T cells’ function [37,38].

On the other hand, to further heighten the uncertainty regarding Treg status in kidney failure, a relatively recent study showed similar frequencies of thymus-derived Tregs present in the circulation of kidney failure patients together with adequate Treg expansion and preserved demethylation status [39]. Interestingly, a meta-analysis of five studies involving kidney failure patients showed that the Tregs-to-CD4^+^ T cells ratio was lower in the non-dialysis kidney failure patients compared to healthy subjects, whereas when both non-dialysis and dialysis kidney failure patients were compared to healthy controls, the difference was lost [40]. These results should most probably be ascribed to the suppressive effect of hemodialysis on all of the CD4^+^ T cell subsets [40]. Furthermore, the dialysis modality itself appears to affect Tregs, with the latter displaying an increasing trend following the start of PD but not hemodialysis [41].

The role of Treg subsets in the induction and maintenance of renal allograft tolerance, the role of Treg subsets in the prediction of kidney transplantation outcomes as well as the indications for the therapeutic use of inducible Tregs in KTRs are under scrutiny [31,42,43]. The impact of the different immunosuppressive regimens on Treg subsets should be taken into consideration in KTRs, with calcineurin inhibitors exerting the most significant effects by reducing Treg survival and their proliferative capacities [44]. Furthermore, the suppressive activity of Tregs in kidney transplantation depends on the presence of specific Treg subsets, including the DR(high+)CD45RA(−) Tregs which represent highly activated mature cells and the activated CD4^+^CD25^+^CD62L^+^CD45RO^+^ Tregs [45,46].

### 2.3. B Lymphocytes

Similarly to T cells, several studies have demonstrated a state of progressive B lymphopenia in CKD which becomes particularly pronounced in kidney failure patients, affecting both the IgM-producing CD5^+^ innate B cells and the CD27^+^ memory B cells [47,48,49]. Notably, the pattern of naïve B cells and transitional CD19^+^CD24^high^CD38^high^ B cell depletion, together with increases in memory (IgD^−^CD27^−^) B cells which are observed in CKD, bear similarities to the aging process [50,51,52]. The triad of decreased bone marrow output, impaired responsiveness to the tumor necrosis factor (TNF) ligand superfamily member 13B (BAFF) and accentuated activation-induced apoptosis of B lymphocytes underlies the pathogenesis of B cell lymphopenia in patients with kidney failure [53]. Both elevated serum levels of B cell-related growth and proliferation factors such as IL-7 and diminished BAFF receptor expression have been observed in patients with kidney failure [50,54]. Furthermore, increased B cell apoptosis, impaired B cell differentiation and maturation as well as deregulation of intrinsic B cell signaling pathways appear to be directly affected by the uremic milieu [50,54].

## 3. The Implication of Cells of the Acquired Immune System in the Pathogenesis of Myocardial Dysfunction in CKD

### 3.1. T Cells

**Figure 1:**
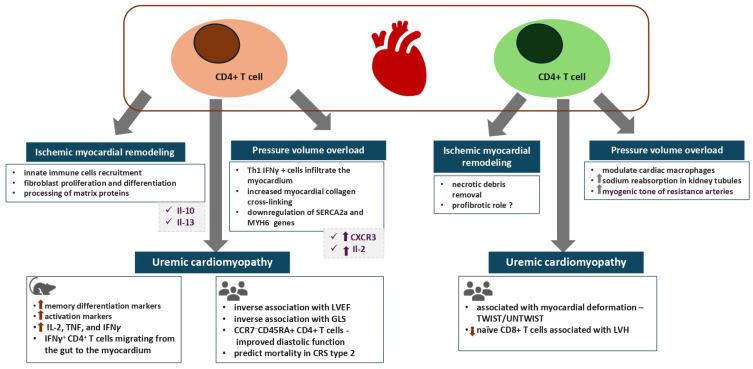
CD4^+^ T cells and CD8^+^ T cells in the pathogenesis of myocardial remodeling and dysfunction in various disease models. CRS2, type 2 cardiorenal syndrome; CCR7, C-C chemokine receptor type 7; CXCR3, chemokine receptor type 3; GLS, global longitudinal strain; IFNγ, interferon gamma; Il, interleukin; LVEF, left ventricular ejection fraction; LVH, left ventricular hypertrophy; Th1, T helper 1 lymphocyte; TNF, tumor necrosis factor; SERCA2a, sarcoplasmic/endoplasmic Reticulum Ca^2+^-ATPase 2a.

The significance of the contribution of T cells to myocardial remodeling in the setting of various disease processes remains to be clarified, as their role in the pathogenesis of myocardial inflammation has only recently come to the center of attention (Figure 1). Evidence from the past 30 years has shed light on the participation of T lymphocytes in myocardial damage due to viral or autoimmune myocarditis, following their activation by antigen-presenting dendritic cells [55]. A considerable body of recent evidence has shown that T lymphocyte subsets are active players in the process of ischemic myocardial remodeling, affecting both the deposition of collagen matrix and fibrosis generation as well as promotion of the myocardial healing processes in this setting [56]. Accordingly, the modulation of cytokine-related pathways, such as Il-10 and Il-13 by CD4^+^ T cells in the myocardium, are suggested to affect innate immune cell recruitment in the myocardium, the proliferation and differentiation of fibroblasts and post-transcriptional processing of matrix proteins [56]. Yet, although CD4^+^ T cells may promote healing of myocardial injury in the acute phase of post-myocardial reperfusion injury, they are involved in the pathogenesis of LV remodeling during the development of ischemic heart failure in the chronic setting.

Experimental data from pressure overload models such as transverse aortic constriction (TAC) have shown that the transfer of T cells into T lymphocyte-deficient mice leads to augmented expression of lysyl oxidases in the myocardium and subsequently increased myocardial collagen cross-linking and fiber density [57,58,59]. In line with the above, active recruitment and augmented adhesion of T cells to the activated vascular endothelial cells, as manifested by upregulated expression of E-selectin, VCAM-1 and ICAM-1, represent prominent features of adaptive immunity activation in pressure overload models [57,60,61,62]. Consequently, myocardial accumulation of T cells and, in particular, effector CD4^+^ T cells with Th-1 polarization occurs, as shown by enhanced expression of interferon γ (IFNγ) and T-bet [57,60,61,62]. The marked expression of the chemokine receptor CXCR3 by Th1 cells appears to be the key mediator which directs their recruitment to the cardiac tissue in response to CXCL9 and CXCL10 chemokines secreted by fibroblasts and innate immune cells in the setting of pressure–volume overload [62,63,64]. Elevated secretion of the T cell growth factor IL-2 after ex vivo TCR stimulation has been observed in TAC-induced heart failure [57]. Moreover, in the same pressure overload models, mice deficient in CD4^+^ T cells display a normal expression of the myocardial relaxation regulator, the sarcoplasmic/endoplasmic reticulum Ca^2+^ ATPase 2a (SERCA2a) as well as the alpha heavy chain subunit of the cardiac myosin MYH6 gene together with lower expression of the natriuretic peptides [57].

Abundant evidence supports the fundamental role of immune mechanisms via pathways involving T cells and their subsets such as the γδ, CD4^+^ and CD8^+^ T cells in the pathogenesis of hypertension, as well as their direct influence on two central regulatory and target organs, such as the kidney and heart [65]. Thus, experimental models of arterial hypertension, another example of a pressure overload stimulus, showed that in rats fed with a high salt diet, kidney tissue infiltration by T lymphocytes was associated with increased expression of NADPH oxidase and amplification of free radical generation [66]. On the other hand, administration of the immunosuppressive medication tacrolimus attenuated all these effects [66]. Furthermore, mice lacking mature T or B cells were protected from myocardial remodeling when submitted to strong hypertensive stimuli such as angiotensin II infusion or administration of deoxycorticosterone acetate salt [67]. On the other hand, adoptive transfer of T cells but not B cells restored the hypertensive responses [67].

Currently, evidence, albeit scarce, points to an intricate role of the CD8^+^ T cells in the development and progression of CVD. Thus, CD8^+^ T cells are inferred to have a profibrotic role in the setting of ischemic myocardial remodeling [68]. Accordingly, post-myocardial infarction mice lacking functional CD8^+^ T cells display delayed removal of necrotic debris and defective scar formation which has been ascribed to proteolytic cleavage of the MerTK tyrosine kinase protein and subsequent inhibition of macrophage-mediated phagocytosis [68]. Available data regarding the role of CD8^+^ T cells in pressure overload models are ambiguous. Thus, CD8^+^ T cells are suggested to modulate the number and activation of cardiac macrophages in a mice model of TAC, even though the relevant mechanisms remain obscure at present [69]. Mice lacking CD8^+^ T cells display an increased number of monocytes and anti-inflammatory M2 macrophages; expressed higher levels of growth factors such as insulin growth factor 1 (IGF1), amphiregulin (Areg) and oncostatin M (ONC); as well as developed protective compensatory myocardial hypertrophy [69].

Regarding the implication of CD8^+^ T cells in arterial hypertension, the existing data are more straightforward. Several lines of evidence have shown that CD8^+^ T cells have a direct impact on the nephron structures via stimulation of the sodium chloride cotransporter leading to increased sodium reabsorption [70]. Accordingly, IFNγ stimulates its receptor expressed within the distal convoluted tubule, inducing increased expression of the CD8-specific antigen presenter MHC-I as well as of programmed death ligand 1 (PDL1) in tubular cells, thus resulting in enhanced interaction between them and the CD8^+^T cells [71,72,73]. Parallel clinical evidence provided from studies in hypertensive subjects has shown increased counts of IFN-γ and of TNF-α-producing CD8^+^ T cells as well as of immunosenescent CD28^null^ or CD57^+^ CD8^+^ T cells in these patients [74,75]. Apart from their effects on tubular function, CD8^+^ T cells also exert effects on the vasculature, as shown by a recent three-dimensional vascular-immune interface model [73]. Accordingly, CD8^+^ T cells from prehypertensive mice increased the myogenic tone of resistance arteries from naïve mice via interaction with smooth muscle cells and modulation of their contractile properties [75]. Notably, increased transcription of several genes linked to the MAPK/ERK pathway, to the lymphocytic response to IFNγ as well as to chemotaxis was observed [73].

Emerging evidence, albeit scarce, is beginning to shed light on the causal implication of T cells in the pathogenesis of uremic cardiomyopathy phenotypes, including LVH, diastolic dysfunction and worsened myocardial strain (Table 1). Accordingly, in an experimental model of uremic cardiomyopathy, CD4^+^ T cells from 5/6 nephrectomized mice displayed increased expression of markers of memory differentiation such as CD44, a cell-surface glycoprotein involved in cell–cell interactions, cell adhesion and migration [76]. In line with the above, the proportion of CD4^+^ T cells expressing activation markers such as PD-1, killer cell lectin-like receptor subfamily G member 1 (KLRG1) and OX40 was increased in mice with CKD [76]. Furthermore, the activated T cells infiltrated the heart tissue within two weeks following establishment of uremic cardiomyopathy as shown by flow cytometric data with heightened expression of genes involved in T cell recruitment, priming and maturation being detected by next-generation RNA sequencing [76]. The most prominent finding of this landmark experimental study was the improvement of myocardial strain indices and diastolic function as assessed by E/A ratio, isovolumic relaxation time and myocardial performance index following depletion of T cells by anti-CD3 antibody injections [76]. Results of a recent study suggest that the gut–immune–heart axis may be pathogenetically involved in the development of diastolic dysfunction in CKD. Thus, mice with CKD which underwent transplantation with gut microbiota from CKD patients displayed an expansion of heart-infiltrating IFNγ^+^ CD4^+^ T cells together with impaired diastolic function [77]. In particular, the Klebsiella pneumoniae quantity in the gut was directly associated with the IFNγ^+^ CD4^+^ T cell counts infiltrating the cardiac tissue [77]. Even though the results of this study indicate that IFNγ^+^ CD4^+^ T cells might proliferate initially in the gut from where they subsequently translocate to the heart, further studies are needed to confirm these findings as well as elucidate the pathways underlying the cardiac tropism of CD4^+^ T cells in this setting.

Thus, taking into consideration the available evidence highlighting the relevance of T lymphocytes in the myocardial fibrotic response, we may infer their beneficial implications during the cardiac repair processes in the setting of ischemia–reperfusion injury and detrimental effects in the setting of chronic pressure overload.

On the other hand, clinical evidence regarding the implication of T cell subsets in the pathogenesis of myocardial remodeling in CKD is scarce (Table 1). Clinical evidence from a small cohort of pediatric patients with CKD revealed that T cell phenotypes correlated with structural and functional echocardiographic indices of myocardial function [76]. Accordingly, increased levels of activated T cells expressing PD-1 and/or CD57 were associated with impaired diastolic function as represented by the E/E’ ratio [76]. On the other hand, the loss of naïve T cells in the same pediatric cohort was associated with exacerbation of LVH [76]. In accordance with the above, in an exploratory study investigating potential associations between the cellular components of the immune system with subclinical indices of myocardial dysfunction in CKD patients and KTRs without established CVD, we found an inverse association of CD4^+^ T cells with left ventricular ejection fraction (EF) in CKD patients as well as with dipyridamole-induced improvements in left ventricular EF in KTRs [78]. Notably, with regard to strain-related indices of left ventricular function in the same cohort of KTRs, CD4^+^ T cells displayed an inverse correlation with dipyridamole-induced improvements in the global longitudinal strain 78 [2]. On the other hand, CKD patients displayed an association of CD8^+^ T cells with indices related to myocardial deformation such as the left ventricular twist and untwist. Notably, left ventricular torsion, as represented by the systolic twist and the diastolic untwist rates, is greater in hypertensive compared to normotensive individuals, which might be ascribed to a compensatory mechanism in the setting of increased aortic stiffness during the early stages of hypertensive cardiomyopathy [79]. Furthermore, augmented left ventricular twist and untwist rates have been reported in asymptomatic CKD patients and appear to be compensatory to impairments in myocardial strain, suggesting a pattern of subendocardial injury, the effects of which are compensated for by the epicardial myocardial fibers [79,80,81]. Thus, in light of the foregoing and considering the implication of CD8^+^ T cells in arterial hypertension, further investigation is needed to shed light on the pathways linking CD8^+^ T cells, arterial hypertension and myocardial remodeling in the setting of CKD.

Alterations in T cells are observed in established heart failure with T cells shifting toward a proinflammatory phenotype expressing TNFα, INF, Il2-, Il-18 and Il-10 together with markers of activation such as CD25 and CD69 [82,83]. Various factors mediate the state of lymphopenia in heart failure including peripheral congestion as well as inflammation and sympathetic activation, otherwise known as the neuro-immuno-hormonal axis [84,85]. Several past studies conducted in patients with stable hear failure have shown that lymphocyte counts may predict survival up to one year following their measurement [86,87,88]. Accordingly, the Seattle Heart Failure Model (SHFM) is a validated prognostic tool of projected survival at baseline and following therapeutic interventions in patients with heart failure [89]. Notably, the score indicates that among several variables, the percentage of peripheral blood lymphocytes is an independent predictor of death in patients with heart failure, whereas strikingly, the incorporation of kidney function added only marginal additional benefit in terms of risk prediction in this model [89]. It is worth mentioning that in a cohort of male patients with cardiorenal syndrome type 2, solely the CD4^+^T cells, among the other lymphocyte subtypes, emerged as independent predictors of mortality in these patients, thus further reinforcing previously presented evidence regarding the deleterious role of the CD4^+^ T cells in HF [90].

**Table 1:** Studies assessing the implication of the acquired immune system cell subsets in CKD-associated myocardial remodeling and dysfunction.

Author/Year	Subjects	Immune Cell Subset	Findings	Notes
Winterberg et al., 2019 [76]	Mice; humans—pediatric patients with CKD	T cells, CD4^+^ T cells, CD8^+^ T cells	Mice Early infiltration of T cells in the myocardium following CKD development; improvements in diastolic function in mice following T cell depletion with anti-CD3 antibody. Pediatric CKD patients PD-1 and/or CD57 ^+^ T cells associated with increased E/E’ in pediatric CKD patients; CCR7^−^CD45RA^+^ CD4^+^ T cells associated with improved diastolic function in pediatric CKD patients; loss of naïve CD4^+^ or CD8^+^ T cells associated with LVH; reduced CD4:CD8 ratio associated with impaired diastolic function in pediatric patients with CKD.	Accumulation of central (CD44^hi^CD62L^+^) and effector (CD44^hi^CD62L^−)^ memory CD4^+^ T cells in spleen and peripheral blood of CKD mice; increased expression of KLRG1, PD-1 and/or OX-40 activation markers in CD4^+^ T cells of CKD mice. Limitations: small sample size.
Han et al., 2023 [77]	Mice; humans—patients with stages 3 to 5 CKD and healthy controls	T cells, CD4^+^ T cells	Augmented myocardial infiltration of CD4^+^ T cells in mice transplanted with gut microbiota from CKD patients; significant increase in cardiac IFNγ^+^ CD4^+^ T cell infiltration in CKD microbiota recipient mice compared to healthy controls; significantly increased cardiac IFNγ^+^ CD4^+^ T cell infiltration associated with more severe diastolic dysfunction in mice administered K. pneumoniae compared to control mice.	Abnormal immune responses induced by aberrant gut microbiome in CKD—a potential link in the gut microbiota–gut–kidney–heart axis; pathways involved in IFNγ^+^ CD4^+^ T cell migration from gut to cardiac tissue in uremic cardiomyopathy unclear.
Duni et al., 2024 [78]	Humans—CKD patients, KTRs without established CVD and healthy controls	T cells, CD4^+^ T cells, CD8^+^ T cells, Tregs	Negative correlation of CD4^+^ T cells with LVEF in CKD patients and with dipyridamole-induced improvement of LVEF in KTRs; CD4^+^ T cells inversely correlated with dipyridamole-induced improvements in GLS in KTRs; independent association of CD8^+^ T cells with improved left ventricular twist and untwist in CKD patients.	Observational study, cross-sectional design; small sample size; expression of immune cell subsets examined in the peripheral blood but not in cardiac tissue.
Duni et al., 2023 [90]	Humans—patients with type 2 CRS and CKD patients without CVD as control subjects	T cells, CD4^+^ T cells, CD8^+^ T cells, Tregs	In Kaplan–Meier analysis, decreased lymphocytes, T lymphocytes, CD4^+^ T cells, CD8^+^ T cells and Tregs associated with mortality at a median follow-up of 30 months (*p* < 0.05 for all log-rank tests) in type 2 CRS patients. In multivariate logistic regression analysis, only the CD4^+^ T lymphocytes were independent predictors of mortality (OR 0.66; 95% CI 0.50–0.87; *p* = 0.004) in type 2 CRS patients.	Inverse association between the CD4^+^/CD8^+^ T cell ratio with proteinuria in CRS-2 patients; decreased Treg levels in patients with CRS-2 vs. CKD patients without CVD; decreased Treg levels in patients with CRS-2 and AF vs. those without AF.
Zhang et al., 2010 [36]	Humans—hemodialysis patients, patients with advanced CKD and healthy subjects as controls	Th17 cells, Tregs	Increased Th17-to-Treg ratio in hemodialysis patients with NYHA III–IV heart failure vs. the NYHA I–II group (3.0:1.9 vs. 1.7:3.2; *p* < 0.01).	Increased serum CRP and IL-6 levels positively correlated with the increased Th17 cells and decreased Tregs.
Vernier et al., 2024 [91]	Mice	T lymphocytes, CD4^+^ T cells, CD8^+^ T cells, B lymphocytes	In type 3 CRS, CD4^+^ T cell and CD8^+^ T cell populations in the kidney mediated renal inflammation and the repair phase of IRI; only B lymphocytes mediated cardiac injury. Significant increase in CD4^+^ and CD8^+^ T cells in the kidneys 15 days after IRI; no differences in cardiac Cd4^+^ T cells and CD8^+^ T cells in cardiac tissue post IRI; B lymphocytes declined in both kidney and cardiac tissue in the setting of renal IRI.	Kidney tissue repair response characterized by Foxp3 activation; cardiac tissue inflammation mediated by IL-17RA and IL-1β.
Lin et al., 2022 [92]	Humans—patients with stage 4 and 5 CKD and non-CKD (controls)	CD19^+^ B cells, CD19^+^CD5^+^ B cells, CD19^+^CD5- B cells	Negative correlation of CD19^+^CD5^+^ B cells with LVDD, LVSD and LVM; LVEF positively correlated with CD19^+^ CD5^+^ B and CD19^+^CD5− B cells; CD19^+^CD5^+^ B cells ≤ 0.03 × 10^9^/L associated with higher risk of all-cause mortality (HR = 2.967, 95%CI: 1.067–8.254, *p* = 0.037).	Retrospective study; cohort of elderly patients; unclarified mechanisms of B cell implications in myocardial remodeling in CKD patients.
Yang et al., 2023 [93]	Humans—kidney failure	CD4^+^ T cells, CD8^+^ T cells, CD19^+^ B cells	Decreased CD3^+^ T cells, CD4^+^ T cells, CD8^+^ T cells and B cells in patients with LVH.	
Molina et al., 2018 [94]	Humans—hemodialysis patients	CD3^+^ T cells, CD4^+^ T cells, CD8^+^ T cells, CD19^+^ B cells	CD19^+^ B cell count < 100 cells/μL at baseline and after 1 year associated with all-cause mortality (HR 2, 95% CI: 1.05–3.8, *p* = 0.03 and HR 3.8, 95% CI: 1.005–14, *p* = 0.04, respectively); CD19^+^ B cell count < 100 cells/μL at baseline associated with CV mortality (HR 4.1, IC 95%: 1.2–14.6, *p* = 0.02).	Prospective observational single-center study; peripheral blood lymphocyte subsets.
Duni et al., 2021 [95]	Humans—PD patients and healthy controls	T cells, CD4^+^ T cells, CD8^+^ T cells, Tregs	Inverse association of the total lymphocyte count and percentage of B cells with overhydration; the percentage of B cells was inversely associated with the presence of CAD.	Small sample size; observational and cross-sectional study; peripheral blood lymphocyte subsets.

CAD, coronary artery disease; CV, cardiovascular; GLS; global longitudinal strain; IFNγ, interferon-gamma; IRI, ischemia and reperfusion injury; KTRs, kidney transplant recipients; LDD, left ventricular diastolic diameter; LVEF, left ventricular ejection fraction; LVH, left ventricular hypertrophy; LVM, left ventricular mass; LVS, left ventricular systolic diameter; PD, peritoneal dialysis; Tregs, T regulatory cells.

### 3.2. T Regulatory Cells

**Figure 2:**
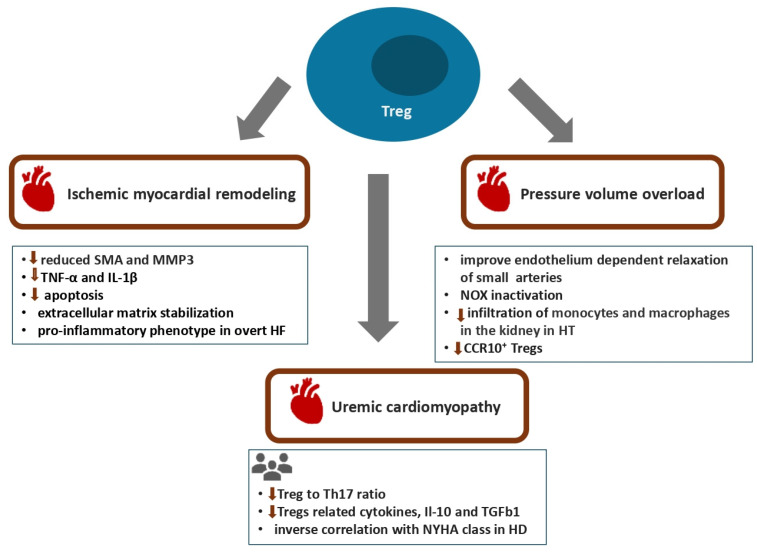
T regulatory cells in the pathogenesis of myocardial remodeling and dysfunction in various disease models. CCR10, C-C chemokine receptor type 10; HD, hemodialysis; HT, hypertension; Il, interleukin; MMP3, matrix metalloproteinase 3; NOX, NADPH oxidase; SMA, smooth muscle actin; TGFb1, transforming growth factor beta 1; Th17, T helper 17 lymphocyte; Treg, T regulatory cells.

Multiple lines of evidence from experimental and clinical studies have highlighted the important role of Tregs in the genesis of myocardial remodeling in the setting of ischemic and hypertensive cardiomyopathy (Figure 2) [96]. Accordingly, Tregs exert direct effects on cardiac fibroblasts, causing reduced expression of smooth muscle actin and matrix metalloproteinase 3 (MMP-3) and eventually leading to extracellular matrix stabilization [97]. In a rat model of acute ischemic heart disease, transfer of Tregs was associated with suppression of myocardial immune infiltrates, reduced expression of TNF-α and IL-1β as well as diminished myocardial fibrosis and cardiomyocyte apoptosis [98,99]. However, at the other end of the spectrum, dysfunctional Tregs with a proinflammatory phenotype accumulate in the setting of overtly established heart failure [100]. These compromised Tregs suppress angiogenesis via both direct effects on the endothelial cells as well as through inhibition of the mobilization and recruitment of angiogenic cells by TNFR1-dependent and C-C chemokine ligand 5/C-C chemokine receptor 5 signaling pathways [100]. Clinical data indicate an association of Tregs with the severity of left ventricular function impairment and that decreased Treg counts might be utilized as an independent adverse prognostic indicator for rehospitalization and reduced survival in patients with heart failure [101]. Accordingly, a ratio of Tregs to CD4^+^ T cells below 6% correlates with an increased incidence of recurrent hospitalization for worsening heart failure [102]. Emerging evidence from both experimental and human models of arterial hypertension supports a critical role for Tregs both in the pathogenesis of hypertension and related target organ damage. Tregs have been shown to improve endothelium-dependent relaxation of the small resistance arteries via IL-10-mediated pathways and to suppress oxidative stress through NOX inactivation [103]. On the other hand, in the setting of Angiotensin II-induced hypertension, expression of NOX2 by CD4^+^CD25^+^FoxP3^+^ Tregs attenuated their suppressive capacity via a reduction in nuclear levels of FoxP3 and NF-κB and eventually led to adverse myocardial remodeling [104]. Moreover, transfer of Tregs to mice treated with saline and aldosterone hindered the infiltration of monocytes and macrophages as well as reduced superoxide levels both in the renal cortex and the aorta [105]. In line with the above, taking into consideration that Tregs express both AT1_B_ and AT2 receptors, transfer of Tregs to angiotensin II-infused hypertensive mice reduced the inflammatory cell infiltrates and TNFα expression in the myocardium, eventually abolishing the development of left ventricular hypertrophy [106,107]. On top of that, transfer of Tregs attenuated the increase in the number of activated and effector memory T cells in the cardiac tissue [106,107]. Hypertensive animals display dysfunctional Tregs characterized by histone deacetylase 6 (HDAC6)-induced deacetylation of Foxp3, which appear to promote apoptosis and myocardial infiltration by inflammatory cells in the setting of ischemia and reperfusion injury [108]. Data from a recent study using pigs with metabolic syndrome and renal artery stenosis indicate that the renoprotective effects of the mesenchymal-cell-derived extracellular vesicles might be at least partially ascribed to the expansion of Tregs [109]. Recent experimental data support a potential linking role of the CD4^+^ T cell-associated enzyme cystathionine γ lyase (CSE) with Tregs and hypertension modulation through the AMP-activated protein kinase-induced differentiation and proliferation of Tregs, which results in suppression of the inflammatory responses in the kidneys and blood vessels [110].

Even though clinical evidence remains limited, mass cytometry studies in hypertensive individuals indicate selective diminution of circulating CCR10^+^ Tregs, which might potentially be associated with impaired respective immunosuppressive mechanisms and accentuated inflammation [111]. There is very little evidence available regarding the role of Tregs in accelerated atherosclerosis and left ventricular remodeling in the uremic milieu (Table 1). A study evaluating the balance between Tregs and Th17 cells and its significance with relation to CVD in patients undergoing maintenance hemodialysis showed that hemodialysis patients, compared to healthy individuals, displayed a reduced Treg-to-Th17 ratio [36]. Furthermore, elevated proinflammatory Th17-related cytokines such as IL-17, IL-6 and IL-23, together with diminished Treg-related cytokines, that is IL-10 and TGFb1, were observed in hemodialysis patients [36]. Notably, hemodialysis patients with NYHA III–IV heart failure exhibited an increase in the Th17-to-Treg cell ratio compared to patients with NYHA I–II heart failure, suggesting that the immune imbalance between proinflammatory Th17 cells and Tregs might mediate myocardial injury in advanced CKD [36].

### 3.3. B Lymphocytes

**Figure 3:**
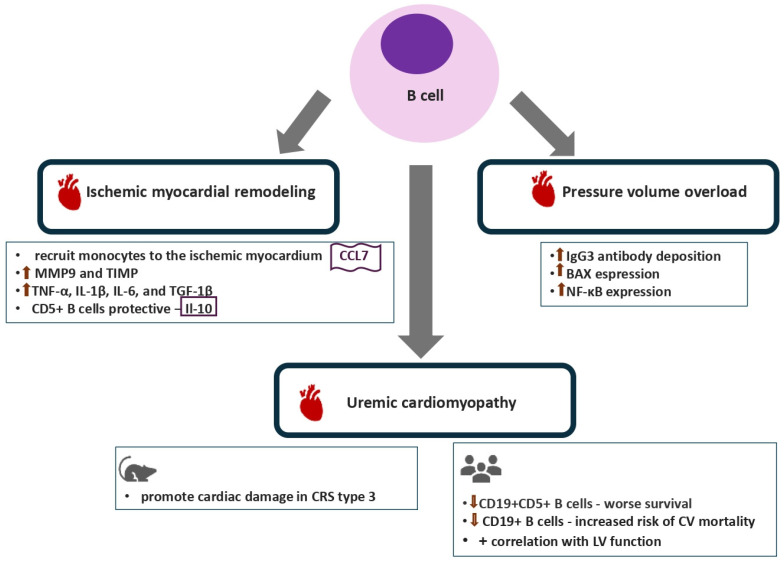
B lymphocytes in the pathogenesis of myocardial remodeling and dysfunction in various disease models. BAX, BCL2-associated X protein; CCL7, chemokine C-C motif ligand 7; CV, cardiovascular; Il, interleukin; LV, left ventricular; MMP9, matrix metalloproteinase 9; NF-κB, neutral factor kappa light chain enhancer of activated B cells; TIMP, tissue inhibitor of matrix metalloproteinase; TGF1β, transforming growth factor beta 1; TNF-α, tumor necrosis factor alpha.

The involvement of B lymphocytes in the development of myocardial remodeling is multifaceted and varies according to the underlying cause of cardiac damage (Figure 3) [112]. Experimental data from animals lacking B lymphocytes support their role in the regulation of both myocardial growth and function [113]. Distinct subpopulations of circulating naïve B cells interact with the endothelium during their transit through the myocardial microvasculature and subsequently accumulate in the cardiac tissue [113]. Myocardial B lymphocytes further influence the immune cell composition in the myocardium as well as the myocardial structure and contractility via pathways involving antigen processing and presentation as well as chemokine signaling [113].

Mature B lymphocytes following activation by TLR-mediated pathways in the setting of myocardial ischemia produce chemokine (C-C motif) ligand 7 (CCL7), a major CCR2 ligand, thus inducing recruitment of monocytes to the area of myocardial infarction and as a result causing further exacerbation of myocardial injury and dysfunction [113]. The recruitment of B cells into the injured myocardium is mediated by the chemokine ligand-receptor CXCL13:CXCR5 axis [114]. On the other hand, B cell deletion in animals with myocardial infarction reduced the expression of mRNA of genes responsible for collagen synthesis and metabolism, such as MMP9 and TIMP, as well as of genes related to the regulation of the expression of proinflammatory cytokines including TNF-α, IL-1β, IL-6 and TGF-1β [115]. Conversely, IL-10-producing CD5^+^ B cells exert an anti-inflammatory and protective role in the setting of myocardial injury, with B cell-specific deletion of IL-10 leading to expansion of myocardial injury and development of myocardial dysfunction [116].

Clinical data indicate that specific B lymphocyte subsets are elevated in heart failure patients, such as TNF-α-positive B lymphocytes, which also correlate with the extent of myocardial fibrosis [115]. Another interesting finding is that increased levels of circulating anti-inflammatory Il-10-producing B regulatory lymphocytes, which are inversely associated with the severity of myocardial dysfunction, have been reported in patients with non-ischemic cardiomyopathy [116].

The pathways underlying the implication of B lymphocytes in the development of hypertensive cardiomyopathy have been elaborated on in an animal model of angiotensin-II-induced heart failure [117]. Accordingly, B lymphocytes promote the expression of inflammatory cytokines, IgG3 antibody deposition and upregulation of Bax expression, a proapoptotic molecule, in the injured myocardium [117]. Furthermore, B lymphocytes induce the augmented expression of nuclear factor kappa B (NF-κB) and collagen deposition by cardiac fibroblasts [117]. Recent research has identified cardiac neoantigens that participate in the induction of antibody-dependent mechanisms in the pathogenesis of myocardial remodeling in pressure overload models [118]. Interestingly, a direct pathogenic role of IgE in the development of cardiomyocyte hypertrophy and cardiac fibroblast activation mediated by the IgE receptor (FcεR1) in the cardiomyocytes has been recently described [119]. Of note, B lymphocytes expressing CD23, a low affinity receptor and negative regulator for IgE, display cardioprotective features in pressure overload models and might become future therapeutic targets [120,121]. On the other hand, B cell-related antibody-independent mechanisms involved in the perpetuation of myocardial injury and dysfunction, such as chemotaxis and activation of inflammatory immune cells or antigen presentation, are a subject of ongoing research [122,123,124].

The potential role of B lymphocytes in the pathogenesis of myocardial dysfunction in CKD and in the development of cardiorenal syndrome has only recently begun to draw attention (Table 1). A study aiming to characterize the cellular immune cell response in the kidney and heart tissue of mice following acute kidney injury (AKI) associated with renal ischemia and reperfusion injury showed that B lymphocytes but not T cells promoted cardiac damage [91]. In addition, a decrease in cardiac B lymphocytes together with a pronounced inflammatory profile in the heart tissue influenced by IL-17RA and IL-1β were observed [91]. A study involving elderly patients with moderate-to-severe CKD showed significantly decreased levels of CD19^+^CD5^+^ B lymphocytes in these patients compared to non-CKD controls [125]. Of note, patients displaying lower levels of B lymphocytes exhibited worse survival [125]. In line with the above, both the CD19^+^CD5^+^ and the CD19^+^CD5- B1 lymphocytes displayed a positive correlation with indices of left ventricular function whereas they were inversely associated with the left ventricular dimensions and mass in elderly patients with advanced CKD [92]. In particular, patients with higher CD19^+^CD5^+^ B lymphocyte levels displayed lower levels of both myocardial dysfunction- and damage-related markers, such as natriuretic peptides and high-sensitivity troponin [92]. On the other hand, despite the established implication of B2 lymphocytes in atherogenesis, there is a paucity of data on their involvement in CVD phenotypes in CKD [126]. Thus, it should be taken into account that the specific roles of B1 and B2 lymphocytes in the development and progression of uremic cardiomyopathy remain to be further clarified by future research. Furthermore, patients with kidney failure who display lower B lymphocyte counts have a higher risk of developing LVH [93]. A prospective study conducted in a cohort of prevalent hemodialysis patients showed that patients with low CD19^+^ B lymphocyte counts carried an increased risk of cardiovascular mortality, including heart-failure-related mortality [94]. With regard to patients undergoing peritoneal dialysis, an inverse association of the percentage of B cells with overhydration indices has been reported [95].

## 4. Future Directions

Development of interventions that modulate the expression and activity of specific immune cell subsets might represent a new target of remarkable therapeutic prospect for uremic cardiomyopathy. Accordingly, the current era of interleukin signaling blockade directed against inflammation in CKD for atherosclerotic CVD has opened doors for new treatments targeting the cellular pathways of the immune system [127]. Thus, trials of anti-CD20 therapy or Treg expansion by low-dose Il-2 in ischemic heart disease are already progressing and shall lay the foundations for their future utilization as an integral part of the already available therapeutic armamentarium in uremic cardiomyopathy [11,128].

## 5. Conclusions

The apprehension of the molecular and cellular immune mechanisms set in motion during the early stages of the myocardial remodeling process in CKD is essential to reverse or at least to hinder its progression to overt heart failure. However, acknowledging the fact that distinct cell subsets of the acquired immune system possess diverse properties with potentially detrimental or alternatively beneficial effects on myocardial remodeling in CKD, the complexity of the molecular signaling pathways in the pathogenesis of uremic cardiomyopathy should be underscored. In addition, it should be acknowledged that current evidence bears limitations, as simultaneous assessment of the changes in lymphocyte subsets in the peripheral blood and cardiac tissue as well as of their functional characteristics is required. Experimental research shall further elucidate and specify the pathophysiological role of immune cell subpopulations, whereas prospective clinical studies are needed to clarify the utility of immune cell populations as potential prognostic markers for the development of uremic cardiomyopathy.

## Data Availability

Not applicable.
